# Translation, Cultural Adaptation, and Psychometric Validation of the Premenstrual Symptoms Screening Tool (PSST) in Serbian

**DOI:** 10.3390/diagnostics16101564

**Published:** 2026-05-21

**Authors:** Dejan Mihajlovic, Momir Dunjic, Nenad Sulovic, Leonida Vitkovic, Kristina Brajovic Car, Radomir Anicic, Jovana Kocic, Nikolia Milijevic, Marija Milic, Jelena Dotlic

**Affiliations:** 1 Department of Obstetrics and Gynecology, Clinical Health Center Kosovska Mitrovica, 38228 Kosovska Mitrovica, Serbia; 2 Department of Obstetrics and Gynecology, Faculty of Medicine, University of Pristina Temporarily Settled in Kosovska Mitrovica, 38228 Kosovska Mitrovica, Serbia; 3 Institute for Histology and Embryology, Faculty of Medicine, University of Pristina Temporarily Settled in Kosovska Mitrovica, 38228 Kosovska Mitrovica, Serbia; 4 The Faculty of Media and Communications, University Singidunum, 11000 Belgrade, Serbia; 5 The Obstetrics and Gynecology Clinic “Narodni Front”, Kraljice Natalije 62, 11000 Belgrade, Serbia; 6 Faculty of Medicine, University of Belgrade, 11000 Belgrade, Serbia; 7 Department of Epidemiology, Faculty of Medicine, University of Pristina Temporarily Settled in Kosovska Mitrovica, 38228 Kosovska Mitrovica, Serbia; 8 Clinic of Obstetrics and Gynecology, University Clinical Center of Serbia, 11000 Belgrade, Serbia

**Keywords:** PSST, premenstrual syndrome, scale validity, scale reliability

## Abstract

**Background/Objectives:** The signs and symptoms of premenstrual syndrome (PMS) can be similar to those of various other illnesses and conditions. To facilitate the detection and categorization of PMS symptoms, specific questionnaires have been developed. The aim of this study was to translate and culturally adapt the Premenstrual Symptoms Screening Tool (PSST) for the Serbian-speaking population and assess its validity and reliability. **Material and Methods:** Our convenience sample included 350 Serbian-speaking female health sciences students from one of the Serbian universities who had PMS symptoms at least once during the past 12 months. They completed a general socio-epidemiologic questionnaire, the PSST, and the Depression, Anxiety and Stress Scale-21 (DASS-21). The PSST was translated according to the recommended methodology, and its psychometric characteristics (internal consistency, construct, discriminant validity and convergent validity) were tested. **Results:** There were no major changes in the items during the process of translation or validation. The Cronbach’s alpha coefficient for the whole scale was 0.927, whereas if an item was deleted, it was >0.8 for all the items. The McDonald’s omega coefficient was 0.896, indicating good internal consistency. The CI–TC coefficients were greater than 0.40 for all the items, indicating that all items were significant elements of the PSST. Exploratory factor analysis extracted three factors. Confirmatory factor analysis revealed adequate values for all model fit estimators. The PSST significantly correlated with the DASS-21, which confirmed its adequate convergent validity. **Conclusions:** The Serbian version of the PSST showed good validity and therefore can be used as a screening tool for PMS in Serbian-speaking young women.

## 1. Introduction

While premenstrual syndrome (PMS) has been common knowledge among gynecologists for decades, its precise cause remains insufficiently elucidated. Moreover, although there are established diagnostic frameworks for PMS, their universal implementation and differentiation from overlapping conditions remain challenging [1,2]. PMS symptoms involve a range of psychophysical manifestations that occur during the luteal phase prior to menstruation and negatively affect the quality of life in many women. The symptoms include irritability, fatigue, sleep disturbances (insomnia or hypersomnia), appetite changes, weight gain, headache, abdominal pain, back or lower back pain, breast tenderness and swelling, joint or muscle pain, bloating, nausea, constipation, and restlessness [1,2]. Experiencing these symptoms can lead to decreased interest in regular activities, such as work or hobbies; difficulty concentrating; lethargy or low energy; and feelings of being overwhelmed or out of control [2,3]. According to the American College of Obstetricians and Gynecologists (ACOG), PMS symptoms are divided into physical/somatic and emotional/psychological categories. The diagnostic criteria for PMS, as defined by the ACOG, include the presence of at least one psychological and one somatic symptom in at least two consecutive cycles [3]. In addition, symptoms should begin five days before the onset of menstrual bleeding, decrease in intensity or completely disappear by the fourth day of menstrual bleeding, not recur until at least the thirteenth day of the menstrual cycle, occur in women who are not undergoing hormone replacement therapy or any therapy for chronic diseases, and not be related to the consumption of drugs or alcohol. Furthermore, these symptoms usually interfere with social, sexual, academic, and occupational functioning [1,3,4]. Diagnosis is even more complicated because the signs and symptoms of PMS can be similar to those of various other illnesses and conditions, such as anxiety, depression, perimenopause, chronic fatigue syndrome, irritable bowel syndrome, and symptoms of thyroid gland diseases [4,5]. Consequently, maintaining a symptom diary across two prospective menstrual cycles is necessary for an accurate PMS diagnosis [3,5].

Data from extant literature indicate that approximately 80% of women worldwide report occasionally having at least one somatic or psychological symptom in the few days prior to menstrual bleeding; however, these symptoms are not regular and mostly do not significantly affect their quality of life. In contrast, approximately 20–40% of women have received a PMS diagnosis, among which 75% have mild complaints. Conversely, approximately 1–8% of women have moderate-to-severe symptoms that can have a significant impact on their quality of life and even their daily functioning [6,7]. In such cases, severe PMS or—according to the Diagnostic and Statistical Manual of Mental Illnesses (DSM-5) classification—a syndrome known as premenstrual dysphoric disorder (PMDD) may be diagnosed [8,9,10]. Although PMS and PMDD share the same pathology, their definitions vary among specialties and in terms of diagnostic classifications. Consequently, the umbrella term “premenstrual disorders (PMD)” has been proposed for an easier representation of both conditions [11]. For reasons of clarity, we utilize the term PMD in this paper.

On account of the complex differential diagnosis, specific questionnaires that facilitate the identification and categorization of PMS symptoms (from mild to severe) have been developed in recent decades. Currently, one of the most commonly utilized questionnaires is the Premenstrual Symptoms Screening Tool (PSST). It is a short and practical screening tool that is useful for everyday practical clinical work with PMD patients. Nevertheless, it should be noted that its findings do not confirm a PMD diagnosis, as this requires recording symptoms over a period of two months [12]. The PSST was developed by Steiner et al. in English and, to date, has been translated and culturally adapted for several populations. However, no translation exists for the Serbian language [12,13,14,15,16,17].

Given the limited number of studies on PMD in Serbia, there is a clear need to validate a scale of this kind for the Serbian context [18]. Such a tool would facilitate PMD diagnosis among Serbian women and, consequently, enable the provision of more effective treatment and support for these women. Additionally, the availability of a validated scale would facilitate a greater number of standardized studies, thus providing more accurate data on the prevalence of this syndrome across different regions of the country and creating opportunities for new scientific insights into PMD. Therefore, the aim of the present study was to translate, culturally adapt, and validate the PSST in the Serbian language to enable the tool to be utilized in the everyday counseling of Serbian-speaking young women.

## 2. Materials and Methods

This cross-sectional study was conducted at the University of Pristina, temporarily seated in Kosovska Mitrovica, over a two-month period (15 April 2023–15 June 2023). Female students from all school years attending health sciences (studies of medicine, dentistry, and nursing) courses were included in this study. Female students were selected for the study as the common proxy for the young and healthy female population. Moreover, according to the extant literature, 46.9% of university students have been diagnosed with PMD, and 10.3% have moderate-to-severe symptoms [19,20]. Before the study commenced, the sample size was calculated (N = 323 respondents, confidence interval = 95%, margin of error = 5%, study power = 95.3%). Study participants were selected as per the following inclusion criteria: age ≥ 18 years, having experienced PMS symptoms at least once during the last 12 months, and being a health sciences student fluent in the Serbian language. The exclusion criteria were refusal to participate, filling out less than 90% of the questionnaire, and suffering from verified specific psychiatric and/or gynecological illnesses which, according to the literature, may be the cause of complaints similar to those of PMS (myoma, adenomyosis, endometriosis, and hormonal disturbances (including polycystic ovary syndrome), anxiety, depression, etc.). Further, other gynecological illnesses (cervical and vulvovaginal pathologies, previous functional ovarian cysts, and previous adnexitis) did not qualify as exclusion criteria because these were not considered to interfere with PMD symptoms. Moreover, the use of hormonal contraception was also not an exclusion criterion if hormones were solely utilized for contraceptive purposes and not for therapeutic ones. All medical conditions were self-reported through a general socio-epidemiological questionnaire. Students were instructed to report only those illnesses that were previously diagnosed (clinically verified and documented in their health records and/or for which they were receiving treatment). The recruitment of respondents was conducted on weekdays during regular compulsory theoretical and practical classes. The response rate was 83.3% (420 approached; six did not fill out the questionnaire; 12 had anxiety or depression; 17 had polycystic ovary syndrome; and 35 declined to participate as they were not interested in responding to surveys or felt tired and opted to take a 15 min break from lectures). Before being included in the study, each student was thoroughly informed about the study and its purpose, after which the student provided her signed consent for participation. The study was approved by the Ethics Committee of the Faculty of Medicine, University of Pristina, temporarily settled in Kosovska Mitrovica (5 April 2023, Approval 09-799).

### 2.1. Instruments

Data from the investigated students were collected via questionnaires. All students completed a general socio-epidemiologic questionnaire, the Depression, Anxiety, and Stress Scale-21 (DASS-21), and the Premenstrual Symptoms Screening Tool (PSST). An agreement for the use of the PSST questionnaire was signed between McMaster University, Hamilton, Canada, and the Faculty of Medicine of the University of Pristina, temporarily settled in Kosovska Mitrovica (license number PSST 23-008).

The general socio-epidemiologic questionnaire included items regarding age, height and weight (to calculate BMI), place of residence before and during schooling, enrolled faculty and year of schooling, general medical and gynecological history, as well as life-style and habits.

The DASS-21 is one of the most reliable and commonly employed instruments designed to assess the severity of general psychological distress and negative emotional states in clinical and nonclinical settings. It is a self-report questionnaire that comprises 21 items/statements divided into three domains (depression, anxiety, and stress), each with seven items/statements. The statements refer to the previous week. The respondents rate each statement from 0 (not at all) to 3 (mostly or almost always). The recommended cutoff values for the severity of mental conditions are ≥10 for the depression scale, ≥8 for the anxiety scale, and ≥15 for the stress scale. The original scores are multiplied by two to obtain the total DASS-21 score. Higher scores indicate a more severe disorder [21].

The PSST is a screening tool that enables the rapid determination of whether a woman has PMS or PMDD according to the DSM-5 criteria, which are the basis of the PSST. It is a useful instrument for revealing moderate-to-severe PMS and PMDD in women with symptoms who would benefit from further diagnostics and therapy [12]. It comprises 19 items/statements divided into two domains; the items are rated on a four-point Likert scale (from absent to severe). The first PSST domain includes 14 items related to physical symptoms (breast tenderness, headaches, joint/muscle pain, bloating, and weight gain), psychological symptoms, and behavioral PMS/PMDD symptoms (anger/irritability; anxiety/tension; tearfulness; depressed mood/hopelessness; decreased interest in work, home, and social activities; difficulty concentrating; fatigue/lack of energy; overeating/food cravings; sleep disturbances; and feeling overwhelmed). The second domain includes five statements measuring the extent to which premenstrual symptoms interfere with a woman’s ability to function (work efficiency or productivity, relationships with coworkers, relationships with family, social life activities, and home responsibilities) [12].

To arrive at a diagnosis of having PMS, the following criteria must be fulfilled according to the diagnostic criteria utilized by the PSST: (1) at least five premenstrual symptoms in the first domain rated as moderate-to-severe, (2) at least one of the first four symptoms rated as moderate or severe, and (3) at least one item in the second domain rated as moderate or severe. The following criteria must be met to establish the existence of PMDD according to the diagnostic criteria used by the PSST: (1) the presence of at least five symptoms from the first domain rated as moderate-to-severe; (2) at least one of the first four core symptoms (anger/irritability, anxiety/tension, tearful/increased sensitivity to rejections, and depressed mood/hopelessness) must be rated as severe; and (3) severe functional impact caused by endorsed premenstrual manifestations [12,13,14,15,16,17].

### 2.2. Process and Adequacy of PSST Translation and Face Validity

The PSST was translated in the standard “forward–backward” manner: (1) one of the investigators, a native Serbian speaker fluent in English, performed the first forward translation from English to Serbian; (2) the second translator, a native English speaker fluent in Serbian, performed the first back translation of the reconciled version; (3) the third investigator, a native Serbian speaker fluent in English, performed the second forward translation from English to Serbian; (4) the fourth translator, a native English speaker also fluent in Serbian, performed the second back translation; and (5) finally, the language coordinator, together with a clinical expert from the team, reviewed all documents and created the final spell-checked version of the PSST in Serbian. All versions of the questionnaire were submitted to McMaster University [12,22].

Further, the pre-final Serbian version of the PSST was tested on 10 female students. The goal of the pilot test was to determine whether any items were confusing, incomprehensible, or culturally inappropriate. All students included in the pilot test were interviewed by the investigating team. To confirm that respondents understood the instructions, questions, and response options, structured interviews were undertaken by the investigating team with all students included in the pilot test. Students and members of the investigating team read through the questionnaire together out loud and discussed the items and answers. All comments made by the students were recorded. The students indicated that there were no issues with any of the items, and they made no suggestions to include additional items. The data obtained during the pilot test were submitted, and approval for the final Serbian questionnaire version was obtained from McMaster University [12].

### 2.3. Statistical Analysis

Descriptive and analytical statistics (percentages, mean values, standard deviations (SDs), and the χ^2^ test) were utilized to briefly illustrate the study population and present the overall PSST scores. Analyses were performed using the SPSS V.20 and JASP V.19.3 programs. To describe the PSST questionnaire, we analyzed the minimal and maximal values, means and standard deviations (SDs), data distributions, and skewness. If the data do not weight toward either of the score extremities, the skewness value should be between −1 and 1 [22].

#### 2.3.1. Internal Consistency

The internal consistency of the Serbian version of the PSST was evaluated using Cronbach’s alpha and McDonald’s omega coefficients. For both coefficients, values of above 0.7 were considered satisfactory [22]. Hotelling’s *t*-square test (HT2) revealed whether there was a significant difference between the obtained mean score values of all PSST items together and the hypothetical case in which the items had equivalent scores [22].

The discriminating characteristics of the questionnaire items were tested via corrected item–total correlation (CI–TC) analysis. This analysis revealed the relationship between one item and the scores of the remaining questionnaire items. An item was considered an adequate part of the questionnaire if the CI–TC was ≥0.40 [22].

#### 2.3.2. Construct Validity

To examine the construct validity of the Serbian PSST, we performed exploratory factor analysis (EFA) and confirmatory factor analysis (CFA). For these analyses, the sample was divided into two equal (n = 175) random groups (one as the EFA sample and one as the CFA sample), as suggested for confirming EFA findings by CFA on a separate sample and to avoid testing the data against itself [21,22]. To obtain reliable questionnaire validation results, it is commonly recommended to include five to ten participants per item [22]. As the PSST had 19 items the sample size of 175 students per group was considered adequate. The Kaiser–Meyer–Olkin (KMO) test and Bartlett’s test of sphericity were utilized to assess the adequacy of sampling (KMO values above 0.7) and determine if the correlations among variables were sufficiently significant for factor analysis (significant *p*-values in Bartlett’s test) [22].

In the EFA, the maximum likelihood factor extraction method was applied to extract the factors. Factor loadings (correlations between the questionnaire items and established factors) were analyzed within the rotated component matrix using a cutoff point of 0.40. To consider an extracted factor as significant, its eigenvalue was required to be above 1.0. Additionally, parallel analysis was performed to ensure that the selection of latent factors was appropriately conducted based on the number of questions in the analysis. Finally, the communality indices (CI—the sum of squared factor loadings) of the questionnaire items were required to be ≥0.4 for an item to remain in the questionnaire [22].

In the CFA with the DWLS estimator, we assessed a few model parameters—such as model significance, goodness-of-fit index (GFI), adjusted goodness-of-fit index (AGFI), comparative fit index (CFI), normative fit index (NFI), parsimony normed fit index (PNFI), relative fit index (RFI), root mean square error of approximation (RMSEA) and standardized root mean square residual (SRMR). RMSEA and SRMR values below 0.08, together with values of other indices above 0.90, indicated a good model fit. The DWLS was chosen as one of the alternative estimators for not normally distributed ordinal data used in small samples [22].

#### 2.3.3. Discriminant Validity

Adequate discriminant validity was considered to be achieved if items related more strongly to their own factor than to others. A correlation coefficient > 0.7 was obtained upon testing the association between an item and its corresponding factor indicated undesired shared variance between factors. In addition, item cross-loadings in the EFA were assessed. Items were required to have factor loadings of above 0.5 for adequate discriminant validity. Finally, the heterotrait–monotrait (HTMT) ratio was calculated using the standard formula. An HTMT value of <0.85 implies appropriate discriminant validity. To exclude common method bias, which may exist in surveys, we performed Harman’s single-factor test and the marker variable technique. If common method bias is an issue, a single factor accounts for the majority of the variance in the model and correlates with a marker variable [22]. For the marker variable (which was not supposed to impact scale scores), we selected students’ height.

#### 2.3.4. Convergent Validity

Convergent validity was investigated using Spearman’s correlation coefficient to weigh the PSST against comparable questionnaires [22]. Numerous studies have revealed relationships among premenstrual disorders, stress, and depression [4]. Therefore, PSST questionnaire scores were correlated with DASS-21 scores. The DASS-21 is one of the most commonly employed instruments for assessing various psychological changes that might occur during the menstrual cycle in certain women [21].

## 3. Results

### 3.1. Description of the Study Sample

Students included in the final sample filled in the complete questionnaire. The study sample incorporated 350 female students who complained of PMD, with a mean +/− SD age of 20.58 +/− 1.97 years, and who were mostly enrolled in the third year of university schooling. The proportion of students studying medicine was 82.9%, dentistry 9.4%, while the remaining 7.7% were nursing students (Table 1). In our sample, the proportion of students who had chronic illnesses (most commonly asthma, n = 37, 10.6%; followed by kidney sand/stones, n = 20, 5.7%; and hypothyreosis n = 20, 5.7%; all other illnesses were registered in fewer than five cases) was 14.6%, while 12.9% had gynecological illnesses. The majority of students in our sample reported having regular menstrual cycles but no previous pregnancies (77.4%). Few students in our sample used contraception (hormonal contraception, n = 40, 11.4%; condom, n = 94, 26.9%; and other methods, n = 6, 1.7%) (Table 1). The investigated students reported that PMS symptoms had mostly been present since the menarche (66.0%), whereas 25% had symptoms during almost all cycles.

### 3.2. PSST Items and Scores

There were no major changes in the items descriptions during the process of translation and validation. A simple literal translation was adequate for almost all PSST items. The average PSST scores according to the items and domains are presented in Table 2. The data were not normally distributed (*p* = 0.001), but skewness and kurtosis were appropriate. The highest average scores were achieved for items #3 and #9 (tearful/increased sensitivity to rejection and fatigue/lack of energy), whereas item #B (relationships with coworkers) had the lowest average score.

In the investigated sample of Serbian-speaking young women, according to the PSST scores and criteria, 161 (46%) had PMSs, whereas 189 (54%) did not have PMSs. On the other hand, the majority of the investigated students did not have PMDD (82.6%).

### 3.3. Internal Consistency

The Cronbach’s alpha coefficient of the Serbian PSST version was 0.927, whereas the McDonald’s omega coefficient was 0.896, indicating good internal consistency. Cronbach’s alpha for the first PSST domain/factor was 0.897, and for the second domain/factor, it was 0.905. The value of the Cronbach’s alpha coefficient if the item was deleted was above 0.80 for all items. The highest coefficient of 0.929 was observed if item #10 was deleted, whereas the lowest Cronbach’s alpha of 0.921 was observed if items #5, #8 and #A were deleted.

There were no significant differences in the frequency of grades per item (1 to 4) in our sample. Nevertheless, there were no items that were only graded with a score of 1 (not at all). This implies that all signs and symptoms assessed by the PSST were present in at least one investigated female student. However, it does not mean that the same individual endorsed all symptoms.

According to Hotelling’s *t*-square test, there was a significant difference between the item scores (HT2 = 915.575; F = 48.388; *p* = 0.001). The values of the CI-TC coefficient for the Serbian PSST version were greater than 0.40 for all items, with the lowest value being 0.408 for item #10 (eating/food cravings). Consequently, all of items were significant elements of the questionnaire.

### 3.4. Construct Validity

As sampling was adequate (KMO = 0.992; Bartlet *p* = 0.001; almost all communalities ≥ 0.6), factor analysis was performed. Through exploratory factor analysis, we obtained three factors (Table 3).

The factor loadings and communality indices after rotation for all items were appropriate (>0.4), indicating a well-defined factor model. The total variance explained by the four extracted factors was 66.15%.

Parallel analysis confirmed the novel three-factor structure, as eigenvalues were higher for the real than the simulated data for the first three extracted factors(*) (Table 4).

In the EFA that we performed, all items that originally formed the second PSST factor were also grouped together (i.e., factor III).

However, for our sample, the 14 items that originally formed the first PSST factor were divided in two groups (group I—items 1 to 3, item 10 and item 14; and group II—items 4 to 9 and 11 to 13). It should be mentioned that some items showed meaningful loadings on both new factors I and II (specifically items #4 and #5). Nevertheless, upon in-depth content analysis of these items (by the study authors, who are obstetrics and gynecology specialists), it was decided that all items were clinically significant and that no items should be removed. Moreover, according to the higher loadings, we opted to keep them in the new factor I together with the majority of items from the original factor I.

When the factor content was thoroughly evaluated, it was seen that group II, i.e., new factor II, incorporated items regarding stress and symptoms, while group I, i.e., new factor I, included items about feeling depressed and lacking energy. Therefore, we proposed names for the two new factors accordingly (low spirit and stress and symptoms).

**Figure 1:**
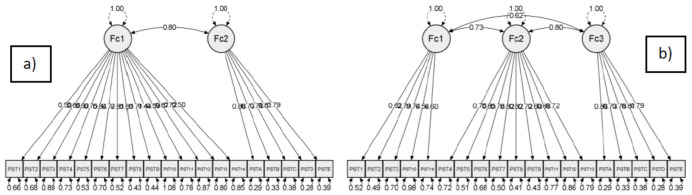
Serbian PSST CFA: (**a**) original two-factor and (**b**) new three-factor construct. Legend: Factor loadings (unstandardized coefficients) are presented on the paths (arrows), and the measurement error variances (residual variances) for each observed variable are presented. The three-factor structure has somewhat lower residual variances, which is considered optimal.

Given that there were discrepancies in the number of original PSST domains/factors and the factors extracted for our examined population, we performed CFA for both the two- and three-factor constructs. For the original two-factor construct, the assessed CFA model fit indices were somewhat lower than optimal. On the other hand, for the new three-factor construct, all values were adequate (Table 5, Figure 1).

Table 6 presents the confirmatory factor analysis factor loadings. As the majority of standardized loadings were relatively high, it can be acknowledged that the items belong to the proposed factors. Nevertheless, this novel fit may partly reflect sample-specific characteristics. Still, as acceptable fit depends on the research context, variables and complexity of the model, and because we considered the distribution of items into two new factors logical (based on the literature and our clinical experience) for our sample, we chose to maintain the novel improved three-factor structure.

### 3.5. Discriminant Validity

After examination of the factor correlation matrix, we observed that the majority of the correlation coefficients between items and factors did not exceed 0.7. Moreover, in the EFA, the factor loadings were above 0.5 for all items except item #4. Finally, the calculated HTMT ratio confirmed adequate discriminant validity (factor 1—factor 2: 0.722; factor 1—factor 3: 0.855; and factor 2—factor 3: 0.798).

We also did not observe common method bias, as the single factor accounted for only 44.62% of the variance. Additionally, PSST scores did not correlate with students’ height (PMS score, *p* = 0.741; PMDD score, *p* = 0.997). As there were no cross-loadings, we concluded that the items demonstrated good construct and discriminant validity.

### 3.6. Convergent Validity

The Serbian version of the PSST displayed adequate convergent validity, as PMS and/or PMDD according to the PSST moderately but significantly correlated with the DASS 21 scores (Table 7).

## 4. Discussion

The present preliminary analysis revealed that the Serbian version of the PSST questionnaire had mostly adequate psychometric properties. In terms of scale properties, the values of the CI–TC coefficient confirmed that they are appropriate aspects of this scale. Moreover, this scale was appropriate for young Serbian women, as they did not find any questions ambiguous or inconvenient. Finally, the study authors (experts in obstetrics and gynecology and epidemiology) considered the questionnaire items/statements adequate and suggested keeping all items/statements in their original form. Future investigations should assess the criterion validity and temporal stability of the Serbian PSST.

The Cronbach’s alpha coefficient of 0.927 for the entire scale indicated excellent internal consistency of the Serbian PSST version. Moreover, all PSST domains also had Cronbach’s alpha coefficients above the acceptable cutoff level. Cronbach’s alpha did not significantly increase when any items were excluded, which confirmed that all items/statements should remain in the questionnaire.

Remarkably good PSST reliability was also proven in other cultural settings [12,13,14,15,16,17]. For example, the Cronbach’s alpha value was 0.91 for Brazilian Portuguese, 0.92 for German and Qatar Arabic, and 0.89 for the Italian language version [12,13,14,15,16,17]. The overall Cronbach’s alpha was higher than that for the individual domains in most previous PSST validations. Additionally, the coefficients for the first domain were higher than those for the second domain [12,13,14,15,16,17]. In contrast, in our setting, the alpha coefficient was greater for the second domain than for the first domain.

Among Serbian-speaking young women, the EFA identified a three-factor structure for the PSST, where the original first domain/factor was divided into two groups of items in accordance with being depressed, under stress, and symptomatic. This structure had better CFA properties than the original construct with two domains/factors. Data from the extant literature also revealed that there were other populations in which factor analysis yielded different domains from that of the original PSST [11,12,13,14,15,16,17]. Italian validation supported a four-dimensional structure of the PSST, whereas validation of the Qatar Arabic language resulted in a five-factor model. The Italian four-factor construct explained 54.3% of the total variance, whereas in our setting, the three-factor construct explained 66.15% of the total variance [17]. In the Qatar Arabic validation, similar to the original study and our validation, one factor incorporated items that interfered with functioning (items/statements A–E). Another factor was the loss of interest in major areas of life [16]. A similar factor was also extracted for the Serbian sample (new factor I). One possible interpretation of these findings is that Arabic and Serbian women understand and value daily functioning and interests in everyday activities differently from other women worldwide. Nevertheless, these assumptions need additional confirmations in further studies. Moreover, physical symptoms and feelings of being overwhelmed are also differently experienced by Arabic women. Therefore, these items appear to be less important for distinguishing PMDD from other types of PMS [14,16]. For Serbian women, being stressed (feeling angry, anxious, and tearful) and consequent overeating formed a specific factor in the Serbian PSST validation, similar to the Qatar Arabic validation (the factor of mood swings). This factor corresponds with one of the PMDD criteria (one of these four symptoms rated as moderate or severe) [12,16].

However, differences in factor structure may have arisen from sampling, translation nuance, context, or statistical decisions, and not necessarily from deep cultural distinctions. It should be noted that the current sample is narrow and specific; thus, the identified structure presents a plausible sample-specific finding that requires replications in larger samples of Serbian women. Consequently, further studies on large samples are needed to assess which factorial structure should be considered valid in populations worldwide, as well as in samples of women with different socio-epidemiologic and general medical characteristics.

Data from the literature reveals associations between the PSST and depression assessment scales, indicating that having a large number of PMS symptoms can increase feelings of depression and negatively impact quality of life. PMDD—with even more intense mental, physical, and psychological symptoms—can even disable normal everyday activities [22,23,24,25,26]. The significant correlation obtained in our study with the DASS-21 indicates that the Serbian PSST can indicate which women might have severe PMS symptoms (particularly psychological symptoms) and which ones are in need of treatment.

Finally, it should be noted that differences in the prevalence of PMSs and PMDD in different populations could also affect the discriminant validity of the PSST, which confirms the need for cultural adaptation and validation across different populations and languages. The occurrence of PMDD in different populations ranges from 3 to 50% [5,24,25]. In our sample, a similar proportion of students had and did not have PMS (approximately 50%), whereas 17.4% had PMDD. These findings correspond to the majority of data from previous studies [19,26], thereby indicating that the Serbian PSST is a suitable instrument.

Our analysis has certain limitations. The applied sampling procedure, with strict inclusion and exclusion criteria, might have caused sampling bias. This survey included only female health sciences students, who are likely to be different from the general population in terms of education, health literacy, stress profile, symptom awareness, and willingness to report symptoms. Consequently, the representativeness of the obtained results is limited for women with lower levels of education or without any medical knowledge. Nevertheless, our sample included a large number of students from various socioeconomic backgrounds, which enables the generalizability of the results to similar student populations. Further studies should be performed to assess the psychometric properties of the PSST in women with different gynecological and psychiatric diseases that have symptoms similar to those of PMS, as such patients were excluded from this study. In addition, future research should address the need for a stratified sample based on age and menopausal status, as the present study is limited with regard to such patients.

Further, it is likely that the inclusion criterion of having PMS symptoms at least once during the last 12 months enriched the sample with symptomatic individuals and narrowed the symptom spectrum. This might have inflated the internal consistency and altered the apparent distribution of responses, causing spectrum-related selection bias. We chose to perform the screening specifically for PMS/PMDD in a population that had any kind of PMS symptoms at least once during the last year, as the PSST is a screening tool. Therefore, we began with the widest possible sample of occasionally symptomatic women to identify those who scored positive on PMS/PMDD screening and should therefore be sent to their gynecologists for further diagnosis and treatment. In addition, from the pilot study performed on the entire population of students at the specific university, we noticed that students who had absolutely no PMS symptoms or any other gynecological issues mostly showed no interest in our study and refused to participate. Nevertheless, we registered that a majority of female students in our settings report having at least a few PMS symptoms occasionally, which corresponds with data from other studies [13,14]. Therefore, we opted to screen for PMD in such students.

Another issue is the fact that the PSST is a retrospective self-reporting screening tool. The retrospective nature of symptom reporting is not adequate for a PMD diagnosis, but only for its screening. Additionally, it might inflate the prevalence of PMD symptoms. This could have been the reason for the higher PMD prevalence in our study compared to data from extant studies. In addition, the data on participants’ health were also only self-reported. Moreover, the PSST was not validated against any existing symptoms checklist. It was validated against DASS-21, which is not a disorder-specific comparator for PMD. However, it is the only available translated and validated tool for the Serbian population that could, at least to a certain extent, provide at least some implications for the mental and emotional states of women with PMD [21]. Because both instruments include emotional and cognitive symptoms, the observed correlations may partially reflect shared symptom content, which should be considered when interpreting the study results.

Another methodological limitation is the absence of a temporal stability assessment, as the test-retest analysis was not performed as part of this study. We tested the same students with PSST (retest), but only after an intervention aimed at reducing PMS symptoms. We plan to report those findings in an upcoming study. Finally, the recruitment occurred during compulsory theoretical and practical classes, which might have made certain students feel a certain amount of pressure to participate.

## 5. Conclusions

The Serbian version of the PSST has adequate validity and therefore appears promising as a screening and PMD symptom assessment tool among Serbian-speaking young women; however, further research on broader and clinically characterized samples is necessary. According to psychiatric and gynecological criteria and recommendations, women who test positive on the PSST screening should undergo prospective assessment of reported symptoms to accurately diagnose PMD and, if needed, receive adequate therapy from a qualified gynecologist.

## Figures and Tables

**Table 1:** Sociodemographic data of the investigated students.

Parameters		Frequency	Percent
Faculty	medicine	290	82.9
	dentistry	33	9.4
	nursing	27	7.7
Year of schooling	1	26	7.4
	2	87	24.9
	3	139	39.7
	4	29	8.3
	5	39	11.1
	6	30	8.6
Gynecological illnesses	no	305	87.1
	yes	45	12.9
Chronic illnesses	no	299	85.4
	yes	51	14.6
Menstrual cycle	regular	312	89.1
	irregular	38	10.9
Previous pregnancies	no	271	77.4
	yes	79	22.6
Hormonal contraception use	no	310	88.6
	yes	40	11.4

**Table 2:** Description of the Serbian PSST.

Items	Mean	Standard Deviation	Skewness	Corrected Item— Total Correlation	Cronbach’s Alpha If Item Deleted
1. Anger/irritability	2.87	0.95	−0.375	0.511	0.925
2. Anxiety/tension	2.63	1.05	−0.195	0.598	0.924
3. Tearful	2.88	1.11	−0.447	0.525	0.925
4. Depressed mood	2.29	1.13	0.257	0.622	0.923
5. Lower interest in work activities	2.48	1.11	0.069	0.703	0.921
6. Lower interest in home activities	2.33	1.13	0.266	0.625	0.923
7. Lower interest in social activities	2.21	1.07	0.318	0.694	0.922
8. Difficulty concentrating	2.26	1.04	0.280	0.729	0.921
9. Fatigue/lack of energy	2.88	0.97	−0.356	0.691	0.922
10. Overeating	2.81	1.12	−0.391	0.408	0.929
11. Insomnia	1.97	1.06	0.652	0.523	0.925
12. Hypersomnia	2.35	1.14	0.196	0.557	0.925
13. Feeling overwhelmed	2.12	1.14	0.517	0.600	0.924
14. Physical symptoms	3.16	1.04	−0.823	0.464	0.927
PST A (impact on productivity)	2.23	1.01	0.413	0.724	0.921
PST B (impact on work relations)	1.76	0.93	0.970	0.661	0.923
PST C (impact on family relations)	1.97	0.99	0.759	0.676	0.922
PST D (impact on social life)	1.91	0.97	0.858	0.704	0.922
PST E (impact on home duties)	2.04	1.01	0.663	0.659	0.922

**Table 3:** Correlation coefficients of PSST items and factors extracted after rotation.

Items	Factor Loadings			Communality Index
	New Factor I (Low Spirit)	New Factor II (Stress and Symptoms)	Factor III (Originally Factor II)	
1. Anger/irritability	0.783	0.845	0.041	0.690
2. Anxiety/tension	0.399	0.574	0.174	0.543
3. Tearful	0.749	0.820	0.156	0.636
4. Depressed mood	0.474	0.437	0.154	0.539
5. Lower interest in work activities	0.869	0.828	0.258	0.821
6. Lower interest in home activities	0.857	0.477	0.142	0.832
7. Lower interest in social activities	0.717	0.680	0.283	0.663
8. Difficulty concentrating	0.733	0.507	0.511	0.649
9. Fatigue/lack of energy	0.722	0.380	0.631	0.642
10. Overeating	0.303	0.739	0.638	0.512
11. Insomnia	0.746	0.187	0.537	0.629
12. Hypersomnia	0.596	0.210	0.386	0.516
13. Feeling overwhelmed	0.680	0.443	0.074	0.618
14. Physical symptoms	0.411	0.660	0.439	0.408
PST A (impact on productivity)	0.136	0.423	0.584	0.659
PST B (impact on work relations)	0.165	0.239	0.844	0.844
PST C (impact on family relations)	0.312	0.182	0.789	0.805
PST D (impact on social life)	0.158	0.256	0.789	0.861
PST E (impact on home duties)	0.03	0.522	0.640	0.703

Legend: Shaded values were considered to belong to the observed factors.

**Table 4:** Parallel analysis results.

Retained Factors Indicated by *	Real Data Component Eigenvalues	Simulated Data Mean Eigenvalues
Factor 1 *	7.439	1.394
Factor 2 *	1.497	1.326
Factor 3 *	1.256	1.194
Factor 4	1.024	1.206
Factor 5	0.884	1.157
Factor 6	0.684	1.116
Factor 7	0.577	1.075
Factor 8	0.574	1.034
Factor 9	0.499	0.993
Factor 10	0.459	0.953
Factor 11	0.422	0.903
Factor 12	0.393	0.870
Factor 13	0.340	0.830
Factor 14	0.326	0.787
Factor 15	0.313	0.749
Factor 16	0.194	0.701
Factor 17	0.181	0.652

**Table 5:** Confirmatory factor analysis of the Serbian PSST.

CFA Model Fit Parameters	Original Two-Factor Construct	New Three-Factor Construct
Significance level—*p* for the CFA model	0.001	0.001
Goodness-of-fit index—GFI	0.749	0.984
Adjusted goodness-of-fit—AGFI	0.773	0.985
Comparative fit index—CFI	0.803	0.987
Normed fit index—NFI	0.773	0.973
Parsimony normed fit index—PNFI	0.776	0.848
Relative fit index—RFI	0.812	0.968
Root mean square error of approximation—RMSEA	0.120	0.052
Standardized root mean square residual—SRMR	0.081	0.066

Legend: CFA fit indices evaluate how well a hypothesized model reproduces the observed covariance matrix. A combination of absolute and incremental indices should be assessed.

**Table 6:** Confirmatory factor analysis factor loadings.

Factors	Items	Standardized Estimates, i.e., Factor Loadings	Lower 95% Confidence Interval	Upper 95% Confidence Interval
New factor I	1. Anger/irritability	0.774	0.707	0.841
	2. Anxiety/tension	0.749	0.570	0.827
	3. Tearful	0.792	0.618	0.866
	10. Overeating	0.527	0.436	0.618
	14. Physical symptoms	0.587	0.392	0.681
New factor II	4. Depressed mood	0.627	0.557	0.696
	5. Lower interest in work activities	0.808	0.765	0.852
	6. Lower interest in home activities	0.842	0.688	0.896
	7. Lower interest in social activities	0.879	0.731	0.927
	8. Difficulty concentrating	0.881	0.733	0.929
	9. Fatigue/lack of energy	0.720	0.664	0.777
	11. Insomnia	0.662	0.484	0.739
	12. Hypersomnia	0.672	0.496	0.748
	13. Feeling overwhelmed	0.677	0.501	0.753
Factor III	PST A (impact on productivity)	0.791	0.632	0.850
	PST B (impact on work relations)	0.889	0.860	0.917
	PST C (impact on family relations)	0.841	0.805	0.877
	PST D (impact on social life)	0.926	0.902	0.949
	PST E (impact on home duties)	0.692	0.633	0.751

Legend: standardized loadings close to 1 or −1 indicate a strong relationship between the factor and the item.

**Table 7:** Correlations of PSST and DASS 21 scores.

PSST and DASS 21 Scores		DASS 21 Total Score	Depression	Anxiety	Stress
PMS score	Spearman’s ρ	0.462	0.410	0.443	0.424
	*p*	0.001	0.001	0.001	0.001
PMDD score	Spearman’s ρ	0.546	0.391	0.329	0.345
	*p*	0.001	0.001	0.001	0.001

## Data Availability

All of the supporting data for the article are presented.
